# TEAD4 modulated LncRNA MNX1-AS1 contributes to gastric cancer progression partly through suppressing BTG2 and activating BCL2

**DOI:** 10.1186/s12943-019-1104-1

**Published:** 2020-01-10

**Authors:** You Shuai, Zhonghua Ma, Weitao Liu, Tao Yu, Changsheng Yan, Hua Jiang, Shengwang Tian, Tongpeng Xu, Yongqian Shu

**Affiliations:** 1 Department of Medical Oncology, The First Affiliated Hospital of Nanjing Medical University, Nanjing, China; 2 Department of Medical Oncology, Jiangsu Cancer Hospital, Jiangsu Institute of Cancer Research, The Affiliated Cancer Hospital of Nanjing Medical University, Nanjing, Jiangsu China; 3 Key Laboratory of Carcinogenesis and Translational Research (Ministry of Education), Division of Gastrointestinal Cancer Translational Research Laboratory, Peking University Cancer Hospital and Institute, Beijing, China; 4 NHC Key Laboratory of Glycoconjugates Research, Department of Biochemistry and Molecular Biology, School of Basic Medical Sciences, Shanghai Medical College of Fudan University, Shanghai, China; 5 Department of Gastroenterology, Institute for Microbial Ecology, School of Medicine, Xiamen University, Zhongshan Hospital, Xiamen University, Xiamen, China; 6 Department of Oncology, The Affiliated Changzhou No.2 People’s Hospital with Nanjing Medical University, Changzhou, 213003 Jiangsu China; 7 Department of Oncology, JinTan People’s Hospital, Jintan, 213200 China

**Keywords:** LncRNA, Gastric cancer, TEAD4, MNX1-AS1

## Abstract

**Background:**

Gastric cancer (GC) is the third leading cause of cancer-related mortality globally. Long noncoding RNAs (lncRNAs) are dysregulated in obvious malignancies including GC and exploring the regulatory mechanisms underlying their expression is an attractive research area. However, these molecular mechanisms require further clarification, especially upstream mechanisms.

**Methods:**

LncRNA MNX1-AS1 expression in GC tissue samples was investigated via microarray analysis and further determined in a cohort of GC tissues via quantitative reverse transcription polymerase chain reaction (qRT-PCR) assays. Cell proliferation and flow cytometry assays were performed to confirm the roles of MNX1-AS1 in GC proliferation, cell cycle regulation, and apoptosis. The influence of MNX1-AS1 on GC cell migration and invasion was explored with Transwell assays. A xenograft tumour model was established to verify the effects of MNX1-AS1 on in vivo tumourigenesis. The TEAD4-involved upstream regulatory mechanism of MNX1-AS1 was explored through ChIP and luciferase reporter assays. The mechanistic model of MNX1-AS1 in regulating gene expression was further detected by subcellular fractionation, FISH, RIP, ChIP and luciferase reporter assays.

**Results:**

It was found that MNX1-AS1 displayed obvious upregulation in GC tissue samples and cell lines, and ectopic expression of MNX1-AS1 predicted poor clinical outcomes for patients with GC. Overexpressed MNX1-AS1 expression promoted proliferation, migration and invasion of GC cells markedly, whereas decreased MNX1-AS1 expression elicited the opposite effects. Consistent with the in vitro results, MNX1-AS1 depletion effectively inhibited the growth of xenograft tumour in vivo. Mechanistically, TEAD4 directly bound the promoter region of MNX1-AS1 and stimulated the transcription of MNX1-AS1. Furthermore, MNX1-AS1 can sponge miR-6785-5p to upregulate the expression of BCL2 in GC cells. Meanwhile, MNX1-AS1 suppressed the transcription of BTG2 by recruiting polycomb repressive complex 2 to BTG2 promoter regions.

**Conclusions:**

Our findings demonstrate that MNX1-AS1 may be able to serve as a prognostic indicator in GC patients and that TEAD4-activatd MNX1-AS1 can promote GC progression through EZH2/BTG2 and miR-6785-5p/BCL2 axes, implicating it as a novel and potent target for the treatment of GC.

## Background

Gastric cancer (GC) is the third leading cause of cancer-related death and the fifth most frequently diagnosed cancer worldwide, with 1,000,000 new gastric cancer cases and 783,000 death predicted in 2018 [1, 2]. Notably, the incidence rates are dramatically increased in Eastern Asia, which has the highest incidence level worldwide in both sexes [2, 3]. The majority of newly diagnosed GC patients have reached an advanced stage due to absence of sensitive and specific biomarkers [4, 5]. Consequently, there is an urgent need to further GC pathogenesis and identify useful markers implicated in GC.

Long non-coding RNAs (lncRNAs) are newly-discovered transcripts larger than 200 nucleotides [6–8]. Recent research has reported that lncRNAs participate in numerous biological activities, such as apoptosis, cell differentiation and metastasis, thus contributing to cancer progression [9–11]. Also, the interplay between lncRNAs and cellular molecules, such as protein, RNA and DNA, can lead to ectopic expression of critical genes [12–15]. Therefore, studies of potential molecules associated with gastric tumourigenesis represent an attempt to identify the possible application of lncRNAs as effective markers in GC.

The novel lncRNA MNX1-AS1 originally identified as a highly-expressed gene in colorectal cancer (CRC) is also known as CCAT5 [16, 17]. Wang et al. demonstrated that CCAT5 can facilitate CRC progression by interacting with STAT3 [18]. Another research by Ye et al. reported that overexpression of MNX1-AS1 is activated by E2F1 in CRC cells and that MNX1-AS1 can increase SEC61A1 expression by sponging miR-218-5p to facilitate CRC development [19]. In addition, CRC patients in high-MNX1-AS1 expression group exhibited remarkably worse prognostic outcome than those in low-MNX1-AS1 expression group, displaying the robust predictive value of MNX1-AS1 in CRC [19]. Mounting evidence has also shown the presence of MNX1-AS1 overexpression in multiple cancers, including glioblastoma, cervical cancer, GC, ovarian cancer and breast cancer [20–23]. It is reported that MNX1-AS1 exerts promoting effects on the proliferative capacities of cancer cells in both glioblastoma and ovarian cancer [20, 21] In breast cancer, MNX1-AS1 has been characterized as an oncogene to induce epithelial-mesenchymal transition (EMT) and activate the AKT/mTOR signal pathways [22]. Moreover, MNX1-AS1 facilitated cervical cancer progression through activating mitogen-activated protein kinase (MAPK) pathway [23]. An earlier research reported by Zhang and his colleagues revealed that increased MNX1-AS1 level predicts poor prognosis in patients with GC [23]. Recently, Ma et al. demonstrated that MNX1-AS1 can exert promotive effects on GC migration and invasion by repressing CDKN1A [24]. However, diverse mechanisms behind MNX1-AS1 dysregulation remain largely unknown in GC. The present study is designed to comprehensively investigate MNX1-AS1-mediated mechanistic models in GC progression.

For the present research, RNA sequencing data from The Cancer Genome Atlas (TCGA) and Gene Expression Omnibus (GEO) datasets were analysed to find deregulated expression of lncRNAs, which were correlated with GC progression. Among the significantly overexpressed lncRNAs, MNX1-AS1 was selected for further investigation, and the results demonstrated that MNX1-AS1 was significantly amplified in GC and its upregulation frequently predicted poor clinical outcomes. Also, TEA domain family member 4 (TEAD4) activated MNX1-AS1 can mediate the expression level of BTG anti-proliferation factor 2 (BTG2) and BCL2 apoptosis regulator (BCL2) in GC cells. Our findings may illuminate the effectors involved in MNX1-AS1 overexpression and further clarify the underlying mechanism by which MNX1-AS1 mediates the malignant phenotype of GC cells.

## Methods

### LncRNA expression profile analysis

Gene expression data of GC were obtained from TCGA and GEO (GSE62254) datasets. The BAM files and normalized probe-level intensity files were downloaded from TCGA and GEO databases. The probe sequences were downloaded from GEO or microarray manufacturers.

### Collection of GC tissues

We collected tissue specimens from 174 GC patients at the People’s Hospital of Jiangsu Province (Nanjing, Jiangsu, China). All the patients signed an informed consents and received no chemotherapy before the operation. 2010 tumour–node–metastasis (TNM) staging recommended by American Joint Committee on Cancer system (AJCC 7th edition) was used to classify tumour stages. This study was approved by the Human Ethics Committee of Nanjing Medical University (Nanjing, Jiangsu, China) and implemented in accordance with the Helsinki Declaration of Principles.

### Cell lines culture

We purchased the MGC803, SGC7901, BGC823, HEK-293 T and GES-1 cell lines from Shanghai Cell Bank Library of the Chinese Academy of Sciences (Shanghai, China). SGC7901, SGC803, GES-1 and HEK-293 T cells were cultured in RPMI DMEM (Invitrogen, Shanghai, China) with 10% foetal bovine serum (FBS) at 37 °C in 5% CO2. BGC823 and GES-1 cells were cultured in RPMI1640 medium supplemented with 10% FBS at 37 °C in 5% CO2.

### RNA extraction and qRT-PCR assays

Total RNA was isolated with TRIZOL reagent (Invitrogen, Grand Island, NY, USA) according to the product descritption. One microliter total of RNA was reverse-transcribed into cDNA using Primescript RT reagent kit (Takara, Dalian, China). cDNA was quantified by RT-PCR and the data were acquired with SYBR Green (Takara, Dalian, China) using Applied Biosystems 7500 instrument. GAPDH was used as an internal control. The primers are listed in Additional file 4: Table S3.

### Cell transfection

The plasmid vectors were purified with a DNA Midiprep kit (Qiagen, Hilden, Germany). Three siRNAs and a scrambled RNA construct were designed to prevent the off-target effects and ensure the efficiency of interference. MiRNA mimics and their corresponding miRNA inhibitors were purchased from Ribobio (Guangzhou, China). Cells were transfected using Lipofectamine 3000 (Invitrogen). The sequences of siRNA and shRNA are presented in Additional file 4: Table S3.

### Cell migration and invasion assays

The details of cell migration and invasion assays are described in Additional file 5.

### Cell proliferation assay

Cell proliferation ability was examined using a Proliferation Reagent Kit I (MTT) (Roche, Basel, Switzerland). Details are available in Supplementary Materials and Methods.

### Flow cytometry assay

We analysed cell cycle and apoptosis using flow cytometry assays based on the manufacturer’s protocol. Details of Flow cytometry assay are supplied in Additional file 5.

### Western blotting

Protein extraction and quantities were poformed according to the description in Additional file 5. All antibodies are listed in Additional file 4: Table S3.

### Tumour formation assay

The programme was approved by the Animal Experimental Ethics Committee of Nanjing Medical University. All experimental procedures involving animals were performed in strict accordance with the NIH Guide for the Care and Use of Laboratory Animals. Details of Tumour formation assay are described in Additional file 5.

### Immunohistochemical (IHC) analysis

For hematoxylin-eosin stain (HE) staining, tumour tissues from mice were embedded and sectioned, and then stained with haematoxylin and eosin. For immunohistochemical studies, we incubated the samples with antibodies against Ki67. After washing the sample with PBS, the second antibody was added. The signal was amplified and visualized with 3′-diaminobenzidine chromogen (DAB), and then counterstained with haematoxylin.

### RNA immunoprecipitation(RIP)

An EZMagna RNA Immunoprecipitation (RIP) Kit (Millipore) was used according to the manufacturer’s protocol. Details of the RIP experiment are obtained in Additional file 5. The detailed information of antibodies are listed in Additional file 4: Table S3.

### Chromatin immunoprecipitation assays(ChIP)

ChIP assays were performed with a MagnaChIP kit (Millipore). The specific method of ChIp is shown in Additional file 5. The primers are listed in Additional file 4: Table S3.

### Luciferase assays

The TEAD4 binding sequence on the promoter region of MNX1-AS1 was determined by JASPAR (http://jaspar.genereg.net/). The details of luciferase assays are described in Additional file 5. Each experiment was repeated three times.

### Fluorescence in situ hybridization (FISH) and subcellular separation

The FISH and subcellular separation assay of SGC-7901 and MGC-803 cells were conducted according to the method described in Additional file 5. The probe sequences are listed in the Additional file 4: Table S3.

### Statistical analysis

The significance of the differences between the groups was evaluated using Student’s t test, Wilcoxon test or χ^2^ test. The data were analysed with SPSS 17.0 software (IBM), and the double-sided *p* value was calculated, *p* values less than 0.05 were considered to indicate a significant difference.

## Results

### LncRNA MNX1-AS1 is significantly overexpressed and functions AS an unfavourable prognostic factor in patients with GC

**Fig. 1:**
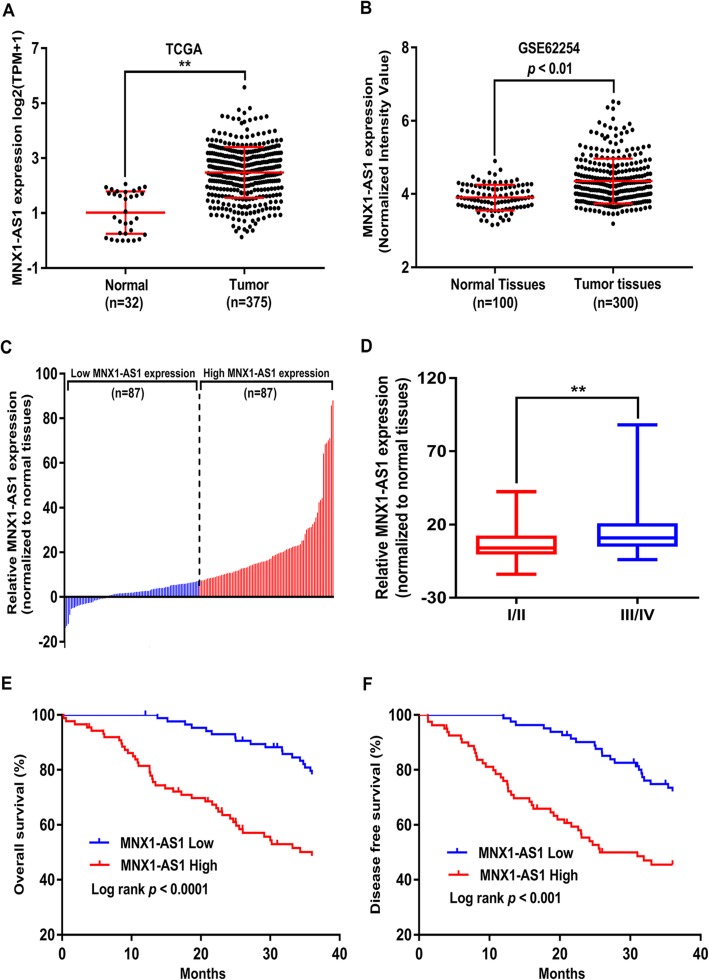
Levels of MNX1-AS1 is significantly increased in gastric cancer tissues and associated with with poor prognosis. **a** Analysis of MNX1-AS1 in GC tissues (*n* = 375) compared with normal tissues(*n* = 32) was analyzed using TCGA data. **b** MNX1-AS1 expression in GC tissues (*n* = 300) and normal tissues (*n* = 100) in the GSE62254 dataset. **c** GC patients were divided into high MNX1-AS1 expression group (*n* = 87) and low MNX1-AS1 expression group (n = 87) according to the median value of MNX1-AS1 expression in GC tissues. **d** The expression of MNX1-AS1 exhibited obvious upregulation in GC patients with a higher pathological stage. **e**, **f** Kaplan-Meier analysis revealed the overall survival (OS) and disease-free survival (DFS) in GC patients based on the relative MNX1-AS1 expression. * *p* < 0.05, ** *p* < 0.01

To detect lncRNAs that potentially drove gastric tumourigenesis, we analysed the RNA-sequencing data from TCGA and GEO datasets (Fig. 1a, b**).** Then, lncRNA MNX1-AS1 was chosen for subsequent research. To determine MNX1-AS1 expression, we performed qRT-PCR assays in 174 GC tissues and matched non-tumour tissues (Fig. 1c). Compared with non-cancerous GC tissues, MNX1-AS1 displayed prominent increase in GC tissue samples. Moreover, a significantly higher MNX1-AS1 expression can be seen in GC cells than in the GES-1 cell line (Additional file 1: Figure S1A). Taken together, these results demonstrate a novel dysregulated lncRNA MNX1-AS1 in GC.

Further, we analysed the relationships between MNX1-AS1 level and clinical factors of GC patients. The high-MNX1-AS1 group (*n* = 87 > median) showed higher tumour stages than the low MNX1-AS1 group (n = 87 < median) (Fig. 1c, d)**.** Additionally, overexpressed MNX1-AS1 expression was obviously associated with tumour size, depth of invasion, histologic grade, TNM stage, lymph node metastasis and distant metastasis in GC patients (Table 1**).**

**Table 1:** Correlation between MNX1-AS1 expression and clinicopathological features of GC

Clinical parameter	*MNX1-AS1 expression*		Chi-squared test *P*-value
	High expression cases (*n* = 87)	Low expression cases (*n* = 87)	
Age (years)			0.263
≤ 50	14	9	
> 50	73	78	
Gender			0.598
Male	67	64	
Female	20	23	
Size			0.004 *
≥ 5 cm	52	33	
< 5 cm	35	54	
Location			0.288
Distal	49	42	
Middle and Proximal	38	45	
Invasion depth			< 0.001 *
T1/T2	9	33	
T3/T4	78	54	
Histologic differentiation			0.035 *
Well and Moderately	28	42	
Poorly	58	45	
TNM Stages			< 0.001 *
I/II	32	63	
III/IV	55	24	
Lymphatic metastasis			0.002 *
Yes	69	50	
No	18	37	
Distant metastasis			0.009 *
Yes	11	2	
No	76	85	

*indicate *P* < 0.05

As shown in Kaplan-Meier survival curve, GC patients in the high-MNX1-AS1 group had markedly shorter overall survival (OS) and disease-free survival (DFS) rates than those in the low-MNX1-AS1 group (Fig. 1e, f)**.** Besides, factors associated with OS and DFS were evaluated using the univariate and multivariate cox regression models. It was found that tumour size, depth of tumour, lymphatic metastasis, TNM stage and MNX1-AS1 expression appeared to correlate with survival period of GC patients (Additional file 2: Table S1, Additional file 3: Table S2). Importantly, multivariate analysis showed that MNX1-AS1 is an independent prognostic factor for worse OS and DFS among GC patients (Additional file 2: Table S1, Additional file 3: Table S2).

### Transcription factor TEAD4 activates lncRNA MNX1-AS1 transcription

**Fig. 2:**
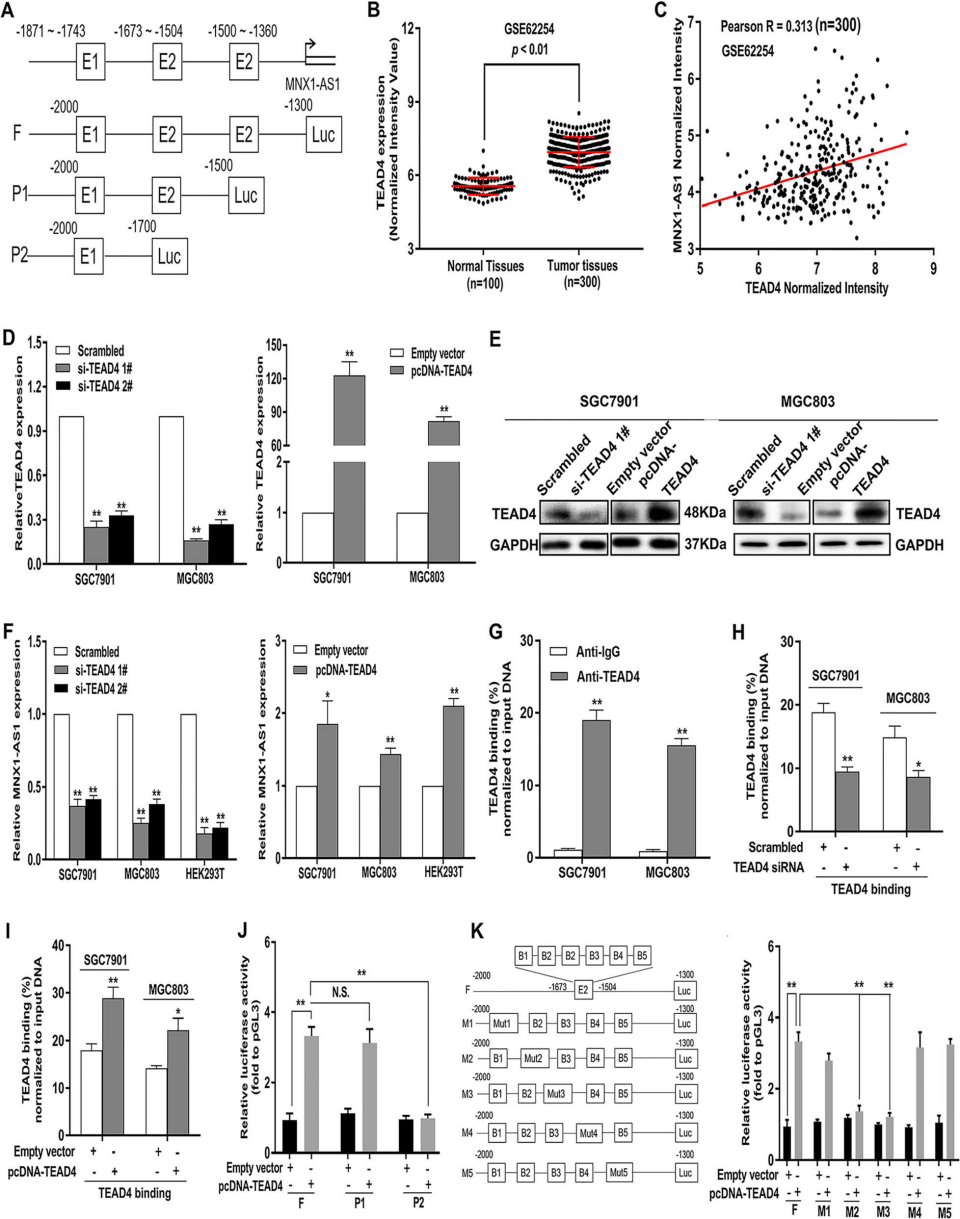
TEAD4 activates MNX1-AS1 expression in GC cells. **a** JASPAR database was used to predict TEAD4 binding site on the promoter region of MNX1-AS1. **b** TEAD4 expression in GC tissues (n = 300) and surrounding tissues (n = 100) in the GSE62254 dataset. **c** The relationship between MNX1-AS1 and TEAD4 was determined by analyzing GSE62254 data. **d** The expression level of TEAD4 in GC cells were determined in GC cells transfected with TEAD4 siRNAs or pcDNA-TEAD4 using qRT-PCR assay. **e** The protein level of TEAD4 was detected in GC cells transfected with TEAD4 siRNAs or pcDNA-TEAD4. **f** MNX1-AS1 expression was determined in GC cells transfected with TEAD4 siRNAs or pcDNA-TEAD4 using qRT-PCR assay. **g** ChIP assays showed endogenous TEAD4 binding to the MNX1-AS1 gene promoter. **h** ChIP assays demonstrated TEAD4 enrichment on the promoter region of MNX1-AS1 with transfection with si-TEAD4. **i** ChIP assays demonstrated TEAD4 enrichment on the promoter region of MNX1-AS1 with transfection with pcDNA-TEAD4. **j** Dual luciferase reporter assays revealed that TEAD4 binds to the E2 promoter region (− 1500 to − 1700) of MNX1-AS1. **k** Dual luciferase reporter assays were performed to determine the exact TEAD4 binding site on the E2 region (− 1500 to − 1700) of MNX1-AS1. * *p* < 0.05, ** *p* < 0.01

Recent research has demonstrated that transcription factors (TFs) can be involved in activating the transcription of some lncRNAs [25–27]. To find the transcription factors closely associated with lncRNA MNX1-AS1 overexpression, we further explored the expression data from the GEO dataset (GSE62254) and conducted a correlation analysis between lncRNA MNX1-AS1 and transcription factors. As shown in Fig. 2b and c, TEAD4 was significantly increased in GC tissues and exhibited a positive correlation with lncRNA MNX1-AS1 in GSE62254 (Fig. 2b, c). Thus, we hypothesized that TEAD4 is involved in the transcription that leads to lncRNA MNX1-AS1 overexpression. To confirm this hypothesis, we made analysis of lncRNA MNX1-AS1 promoter using the JASPAR algorithm and found TEAD4-binding site regions (Fig. 2a).

To clarify the upstream regulatory mechanism, we conducted chromatin immunoprecipitation (ChIP) assays and found that TEAD4 directly interacted with the TEAD4 binding sites within the MNX1-AS1 promoter in SGC7901 and MGC803 cells (Fig. 2g)**.** Next, we performed loss- and gain-of-function assays, and detected that the knockdown or overexpression (Fig. 2d, e), decreased or increased TEAD4 enrichment on the MNX1-AS1 promoter respectively (Fig. 2i, h), contributing to MNX1-AS1 downregulation or upregulation in GC cells, respectively (Fig. 2f)**.**

As shown in Fig. 2a, three promoter regions, designated as Luc-MNX1-AS1-F (− 1300 to − 2000), Luc-MNX1-AS1-P1 (− 1500 to − 2000), and Luc-MNX1-AS1-P2 (− 1700 to − 2000), were cloned into a luciferase reporter plasmid to ensure the TEAD4-binding sites (Fig. 2a). Dual-luciferase reporter assays revealed that TEAD4 binds to the E2 (− 1500 to − 1700) region (Fig. 2j). Then, we cloned the E2 promoter region of MNX1-AS1 into a luciferase reporter plasmid and respectively made mutations at 5 putative binding sites to further determine the exact binding sites. HEK293T cells were co-transfected with the pcDNA-TEAD4/Empty-vector and Luc-F, Luc-M1, Luc-M2, Luc-M3, Luc-M4, or Luc-M5. Both Luc-M2 and Luc-M3 were found to markedly reduce promoter activity compared with Luc-F, Luc-M1, Luc-M4, and Luc-M5 (Fig. 2k)**.** These results demonstrated that the TEAD4-binding site cluster is responsible for lncRNA MNX1-AS1 transcription.

### lncRNA MNX1-AS1 enhances GC cell proliferation, migration, and invasion and inhibits apoptosis

**Fig. 3:**
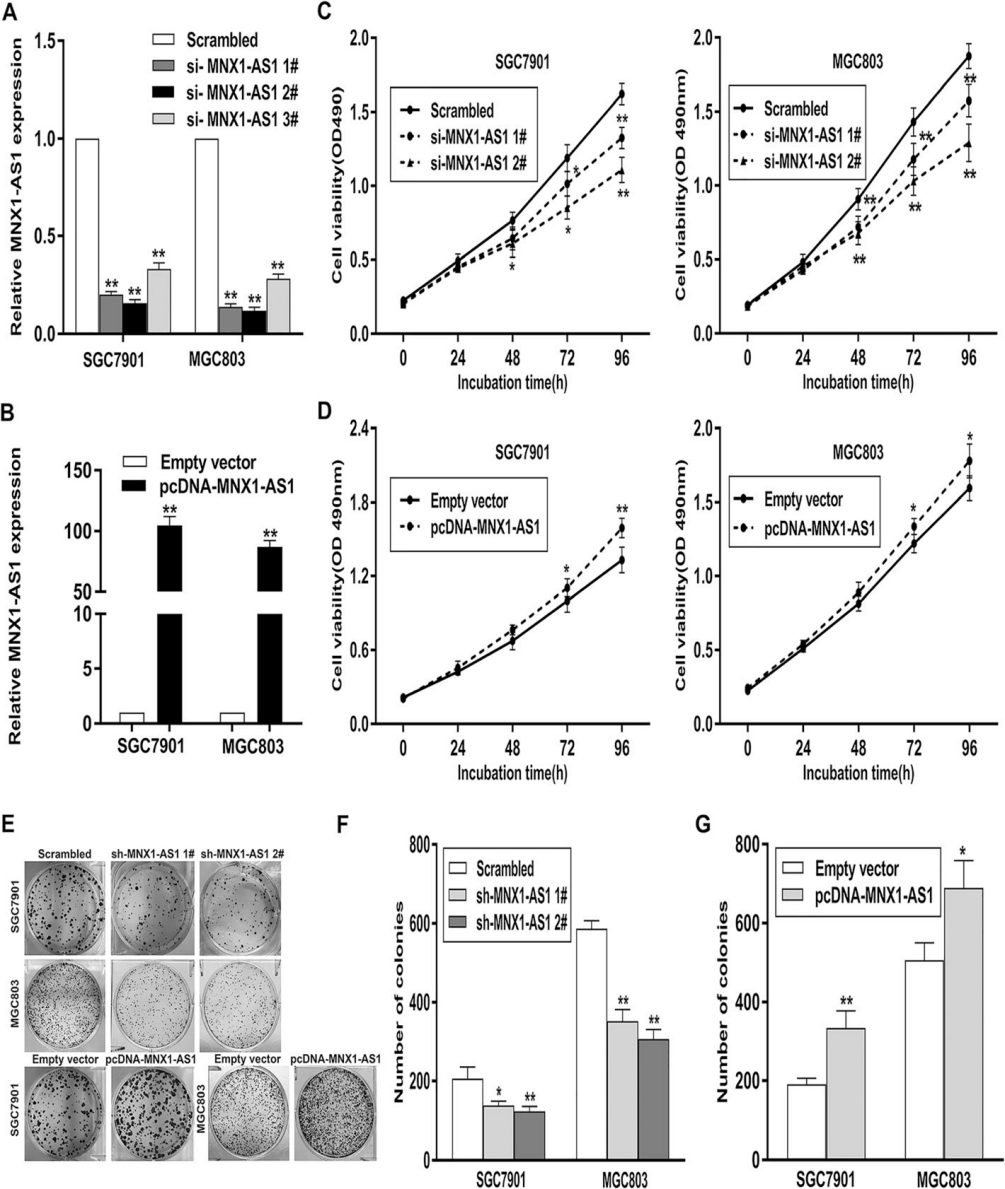
MNX1-AS1 promotes cell proliferation in GC. **a**, **b** qRT-PCR analysis of MNX1-AS1 expression in GC cells transfected with MNX1-AS1 siRNAs or pcDNA-MNX1-AS1. **c**, **d** MTT assays were performed to determine the cell viability of GC cells transfected with si-MNX1-AS1or pcDNA-MNX1-AS1. **e**, **f**. Colony-forming assays were performed to determine the colon-formation ability of GC cells transfected with sh-MNX1-AS1or pcDNA-MNX1-AS1. * *p* < 0.05, ** *p* < 0.01

To clarify the effects of lncRNA MNX1-AS1 dysregulation on GC cell phenotype, we conducted loss-of function and gain-of function assays in GC cells (Fig. 3a, b)**.** MTT and colony formation assays indicated that knockdown of lncRNA MNX1-AS1 impaired GC cell proliferation, whereas overexpression of lncRNA MNX1-AS1 elicited opposite effects (Fig. 3c-g).

**Fig. 4:**
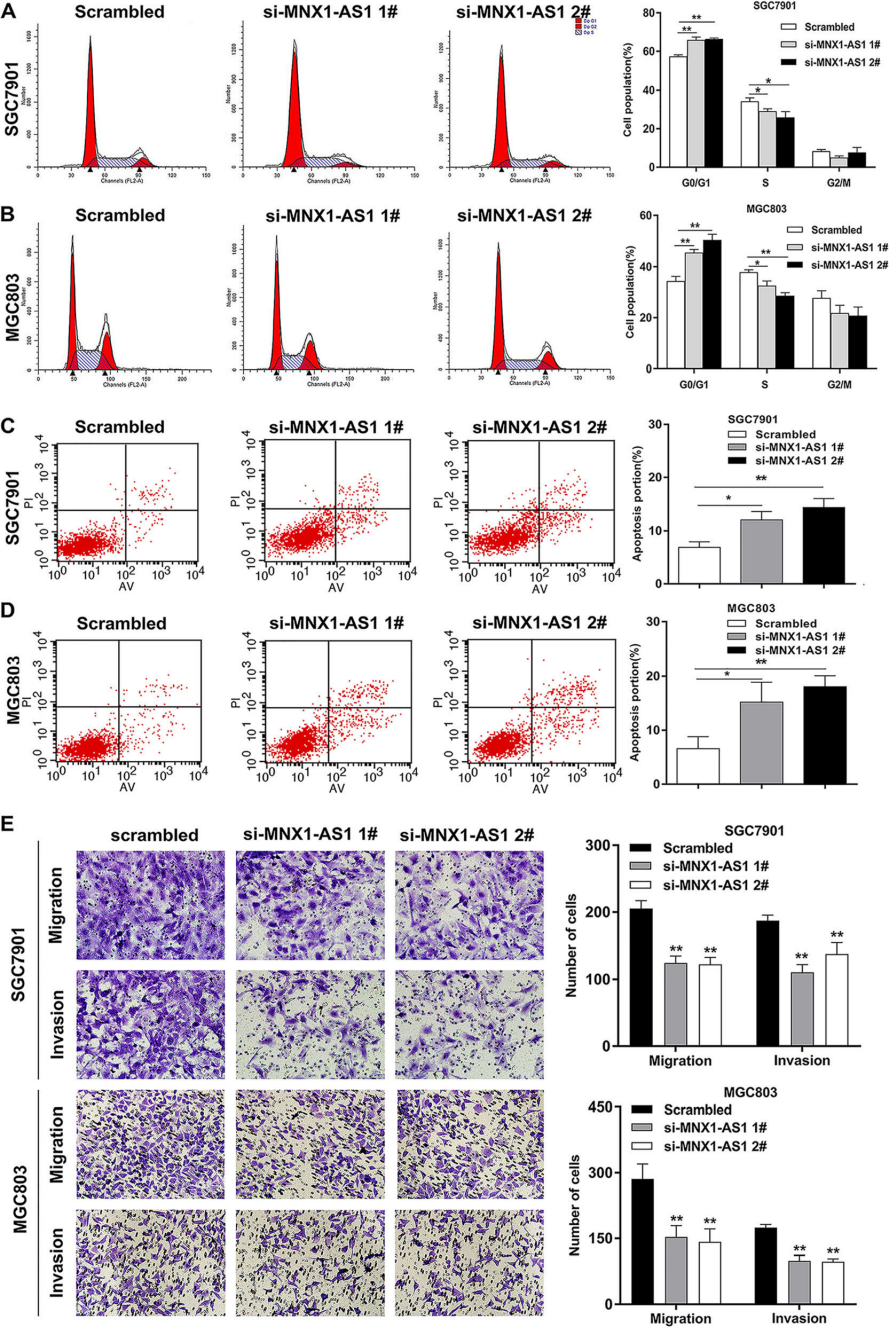
MNX1-AS1 induces cell cycle regulation and apoptosis, and promotes migration and invasion in GC. **a**, **b** Cell cycle analysis showed the effects of MNX1-AS1 on GC cell cycle regulation. **c**, **d** FACS analysis of the effects of MNX1-AS1 on GC cell apoptosis. **e** Transwell assays found that MNX1-AS1 knockdown suppressed GC cell migration and invasion

To determine the effects of lncRNA MNX1-AS1 on cell cycle regulation, flow cytometry assays were conducted in GC cells. An obvious increase in the G1/G0 phase population of MNX1-AS1-depleted GC cells was observed compared with the respective control groups (Fig. 4a, b). Moreover, SGC7901 and MGC803 cells with MNX1-AS1 knockdown exhibited higher levels of apoptosis than control group cells (Fig. 4c, d)**.** Additionally, Transwell assays indicated that the suppression of MNX1-AS1 attenuated the invasive and migratory capacities of GC cells (Fig. 4e)**.** Conversely, overexpression of MNX1-AS1 exhibited promotion effects on the invasive and migratory capacities of GC cells (Additional file 1: Figure S1B). These findings revealed that lncRNA MNX1-AS1 enhances GC cell proliferation, migration and invasion, induces G1/G0 phase arrest, and activates apoptosis, thus contributing to GC progression.

### Inhibition of lncRNA MNX1-AS1 suppressed GC tumourigenesis in vivo

**Fig. 5:**
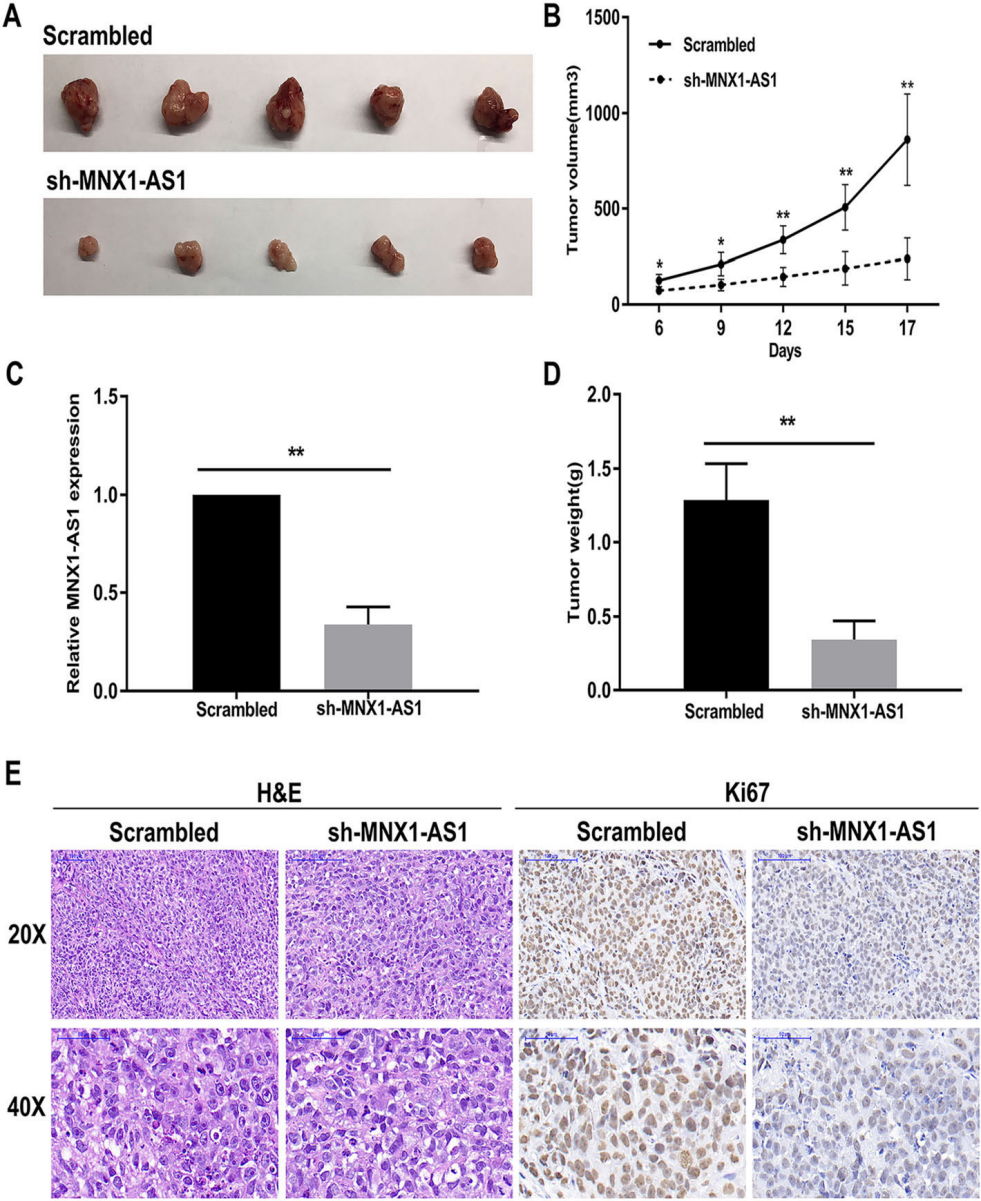
Effect of MNX1-AS1 on GC cell growth in vivo. **a** The images of dissected tumours from MGC803 cells stably transfected with scrambled or sh-MNX1-AS1. **b** The tumour volumes were measured every 3 days after inoculation. **c** The expression levels of MNX1-AS1 in dissected tumour tissues were detected by qRT-PCR assay. **d** The tumour weights were measured and recorded after the tumours were harvested. **e** Tumour tissue samples were immunostained for H&E and Ki-67. *P < 0.05 and **P < 0.01

To explore whether lncRNA MNX1-AS1 affects tumour growth in vivo, we injected lncRNA MNX1-AS1 stable knockdown MGC803 cells, or control cells into male nude mice and constructed a xenograft tumour model. As shown in Fig. 5a, tumours formed in the sh-MNX1-AS1 group were dramatically smaller than those formed in the control group (Fig. 5a). In addition, the efficient knockdown of MNX1-AS1 significantly decreased tumour volume and weight compared with control group (Fig. 5b, c, d)**.** Moreover, immunohistochemistry (IHC) staining assays revealed that lncRNA MNX1-AS1 knockdown group had lower ki-67 protein levels (Fig. 5 e). These findings indicated that lncRNA MNX1-AS1 may inhibit tumour growth in vivo.

### lncRNA MNX1-AS1 mediated GC progression by repressing the expression of BTG2

**Fig. 6:**
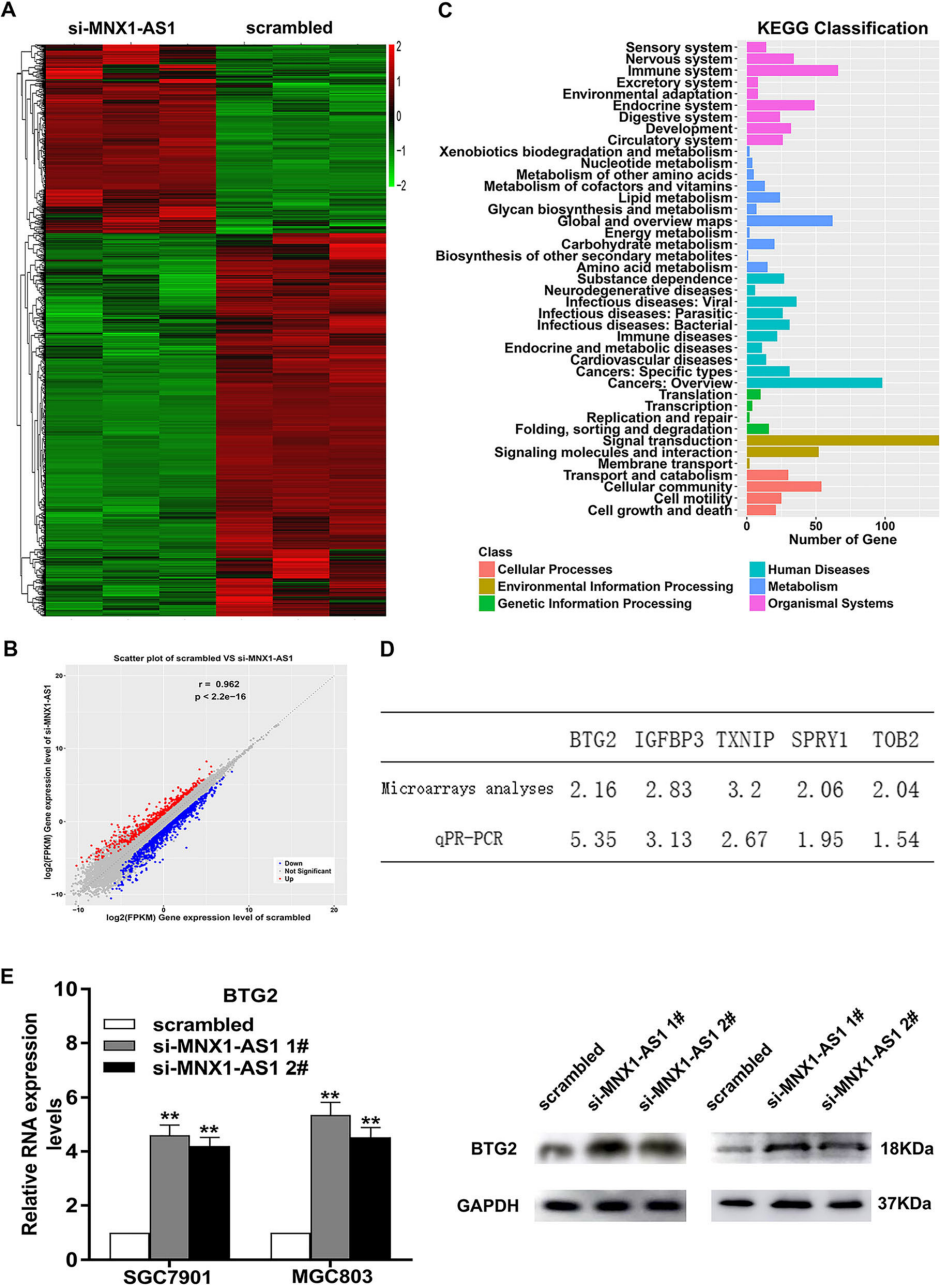
BTG2 is a key downstream target of MNX1-AS1 in GC. **a** The scatter plot of RNA transcription sequencing of the control group and the si-MNX1-AS1 group. **b** Hierarchical clustering gene transcription altered (with fold change greater than 1.5) after knockdown of MNX1-AS1 in SGC7901 cells. **c** GO analysis for all altered genes after knockdown of MNX1-AS1. **d** The qRT-PCR assays were used to selectively examine the alteration of several genes involved in GC progression. **e** BTG2 showed obvious increase in GC cells with MNX1-AS1 knockdwon at both mRNA and protein levels

To further explore the molecular mechanism about how lncRNA MNX1-AS1 contribute to the progression phenotype of GC cells, we performed RNA-sequencing assays from control or siRNAs against MNX1-AS1 in SGC7901 cells (Fig. 6a, b). GO analysis demonstrated that the potential targets of MNX1-AS1 were involved in various biological processes, such as signal transduction, cell growth and death (Fig. 6c). Then, qRT-PCR assays were used to confirm the changes in the level of possible targets mediated by MNX1-AS1 (Fig. 6d). We chose BTG2 for subsequent research, knowing that BTG2 exhibited the highest expression level in MNX1-AS1-knockdown cells (Fig. 6d)**.** As shown in Fig. 6e, BTG2 was obviously increased in MNX1-AS1 depleted GC cells at mRNA and protein levels (Fig. 6e).

**Fig. 7:**
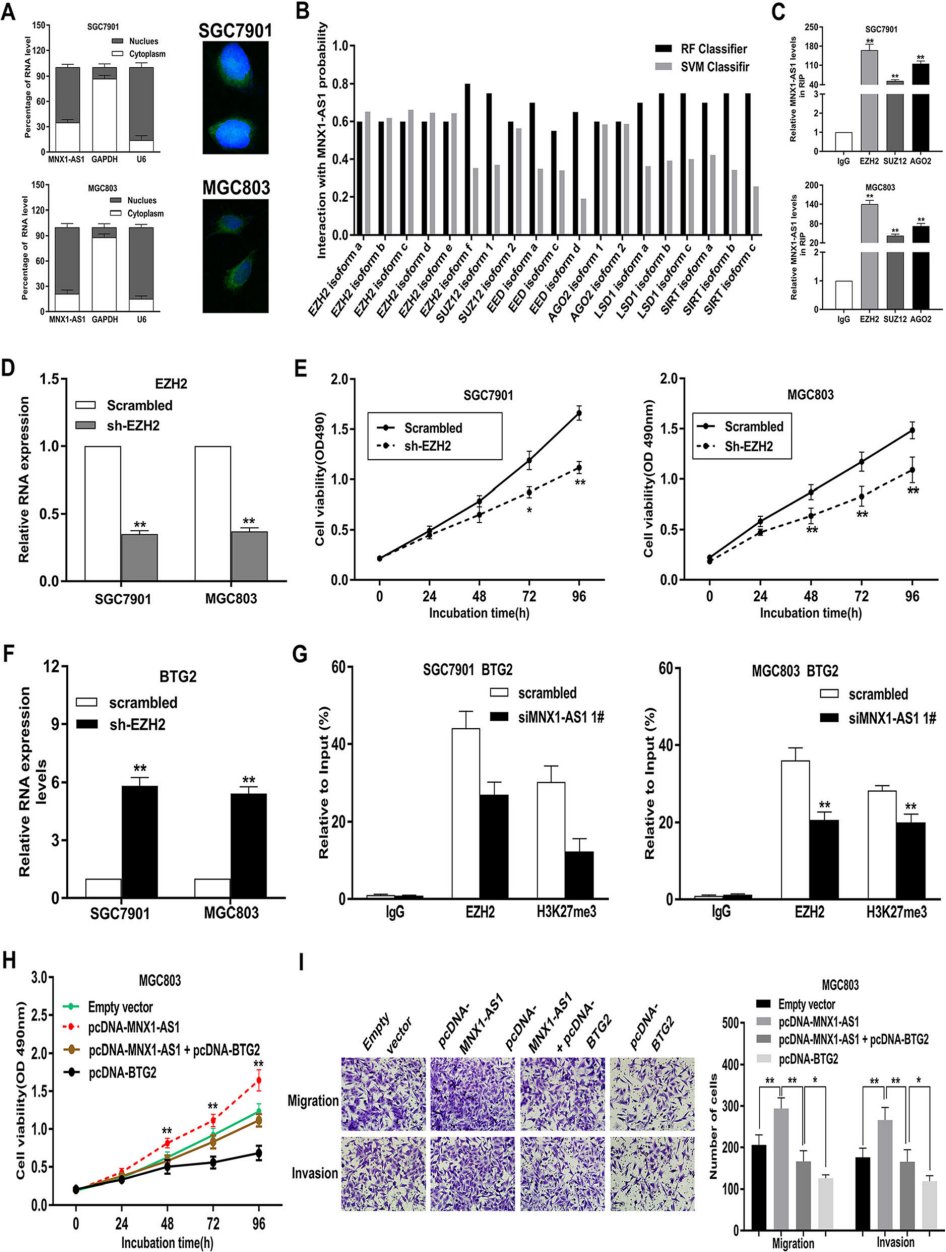
lncRNA MNX1-AS1 suppresses BTG2 transcription by binding to EZH2. **a** FISH and subcellular fractionation assays were used to determine the distribution of lncRNA MNX1-AS1 in SGC7901 and MGC803 cells. **b** Bioinformatics analysis was used to predict RBPs interacting with MNX1-AS1. **c** The RIP assays revealed the fold enrichment of MNX1-AS1 in EZH2/SUZ12/AGO2 RIP, as compared with its matched IgG control. **d** EZH2 was significantly decreased in GC cells transfected with EZH2 shRNA compared with scrambled group. **e** MTT assays were conducted to test the effects of EZH2 dwonregulation on GC cell viability. **f** EZH2 knockdown could obviously increase BTG2 expression in SGC7901 and MGC803 cells. **g** ChIP–qPCR analysis of EZH2 occupancy and H3K27me3 binding on the BTG2 promoter region after knockdown of MNX1-AS1 in SGC7901 and MGC803 cells. IgG was used as a negative control. **h**, **i** The promotion of GC proliferation, migration and invasion was partially reversed by overexpressing BTG2 expression in MGC803 cells

To further clarify the regulatory mode of MNX1-AS1 in GC, we performed FISH and subcellular fractionation assays to study MNX1-AS1 distribution in GC cells. It was shown that MNX1-AS1 mainly localized in the nucleus, implying the role of MNX1-AS1 in transcriptional processing (Fig. 7a). Current evidence has revealed that lncRNAs contribute to cancer phenotypes by interacting with RNA-binding proteins (RBPs) [27–30]. Interestingly, the prediction results by bioinformatics indicate possible binding between MNX1-AS1 and RBPs, such as Enhancer of zeste 2 (EZH2), SUZ12, EED, AGO2, LSD1 and SIRT (http://pridb.gdcb.iastate.edu//RPISeq/) (Fig. 7b). Subsequently, RNA immunoprecipitation (RIP) experiments demonstrated that MNX1-AS1 bound with polycomb repressive complex 2 (PRC2) and argonaute 2 (Ago2) in both SGC7901 and MGC803 cells (Fig. 7c).

EZH2, a critical member of PRC2, catalyses the demethylation and trimethylation of H3 lysine 27 trimethylation (H3K27me3), thus suppressing expression levels at the transcriptional levels [31, 32]. As shown in Fig. 7d and e, depletion of EZH2 markedly inhibited GC cell viability (Fig. 7d, e**).** Importantly, BTG2 was remarkably upregulated in EZH2-depleted SGC7901 and MGC803 cells, suggesting that BTG2 may become a prospective downstream target induced by MNX1-AS1 in GC cells (Fig. 7f**)**. To further study whether lncRNA MNX1-AS1 modulates transcription repression of BTG2 through EZH2-mediated H3K27me3, we performed ChIP assays and found that EZH2 bound the promoter region of BTG2 and induced H3K27me3 modification in GC cells. In addition, the binding activity and H3K27me3 levels were obviously decreased in MNX1-AS1-depleted GC cells than those in control cells (Fig. 7g). These findings revealed that lncRNA MNX1-AS1 can suppress the expression level of BTG2 through EZH2-mediated H3K27me3 in GC.

Moreover, rescue experiments were used to study BTG2 involvement in MNX1-AS1-induced contributions to GC cell proliferation, migration and invasion. Therefore, we performed cotransfection in MGC803 cells to reach the purpose of upregulating both MNX1-AS1 and BTG2. MTT assays showed that the cotransfection could partly reverse pcDNA-MNX1-AS1-induced growth (Fig. 7h). The results of transwell experiments revealed that cotransfection with pcDNA-MNX1-AS1 and pcDNA-BTG2 could significantly rescued pcDNA-MNX1-AS1-mediated promoting effects on GC cell invasion and migration (Fig. 7i).

### LncRNA MNX1-AS1 functions AS a ceRNA and sponges miR-6785-5p in GC cells

Previous studies have indicated that lncRNAs exert critical roles in regulating gene expression by acting as ceRNAs for miRNAs [33, 34]. As shown in Fig. 7a, approximately 35% of lncRNA MNX1-AS1 was distributed in the cytoplasm in SGC7901 cells and about 21% of MNX1-AS1 was distributed in the cytoplasm in MGC803 cells (Fig. 7a). These findings suggest that lncRNA MNX1-AS1 might also have a post-transcriptional regulation function that contributes to GC progression.

**Fig. 8:**
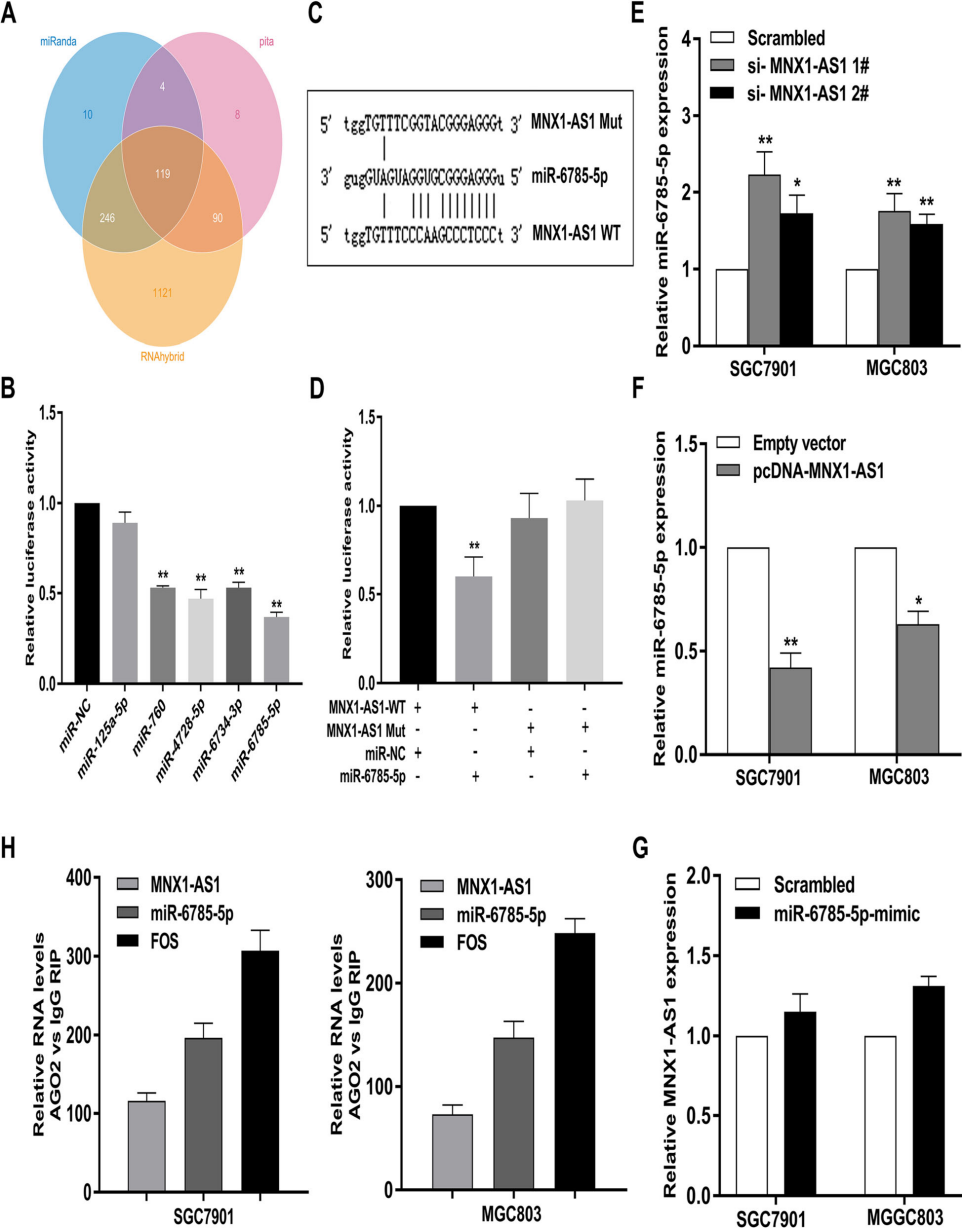
Regulation relationship between MNX1-AS1 and miR-6785-5p. **a** Prediction of potential miRNAs targeting lncRNA MNX1-AS1 were analyzed using miRanda, pita, and RNAhybrid databases. **b**-**d** The luciferase reporter plasmid (pMIR-GLO-MNX1-AS1) was cotransfected into HEK-293 T cells with 5 miRNA-coding plasmids. The interacting activity between miRNAs and MNX1-AS1 was clarified using luciferase reporter assays. **e**, **f** MNX1-AS1 knockdown could obviously upregulate miR-6785-5p in GC cells, and MNX1-AS1 overexpression could significantly downregulate miR-6785-5p. **g** No significant difference in MNX1-AS1 expression after overexpression of miR-6785-5p in GC cells. **h** RNA levels in immunoprecipitates are presented as fold enrichment in AGO2 relative to IgG immunoprecipitates in SGC7901 and MGC803 cells

To confirm our hypothesis, we performed RIP assays in GC cells and found that MNX1-AS1 could directly interact with Ago2 (Fig. 7c), which has been identified as a critical member of RNA-induced silencing complex (RISC) and closely correlates with mRNA repression mediated by miRNA. Then, we predicted potential miRNA targeting sites on lncRNA MNX1-AS1 using miRanda, pita, and RNAhybrid (Fig. 8a). Among the miRNAs, we mainly focus on miR-125a-5p [35, 36], miR-760 [37, 38], miR-4728-5p [39, 40] and miR-6785-5p [40], knowing that they are closely correlated with other malignancies.

To determine the interaction between lncRNA MNX1-AS1 and the listed miRNAs, we performed dual-luciferase reporter assays in HEK-293 T cells transfected with a luciferase plasmid harbouring the sequence of MNX1-AS1 together with plasmids encoding the miRNAs or a control sequence. The results revealed that miR-760, miR-4728-5p and miR-6785-5p significantly inhibited luciferase activity driven by lncRNA MNX1-AS1, and among the listed miRNAs, the suppression ability of miR-6785-5p was the strongest (Fig. 7b)**.** Thus, luciferase reporter constructs were generated to detect binding between MNX1-AS1 and miR-6785-5p (Fig. 8c). It can be observed that the luciferase activities were attenuated in HEK293T cells cotransfected with miR-6785-5p and MNX1-AS1-wile-type (MNX1-AS1-WT), but not the MNX1-AS1-mutation (MNX1-AS1-Mut) (Fig. 8d).

Moreover, qRT-PCR assays showed that miR-6785-5p expression was obviously increased in MNX1-AS1-depleted GC cells (Fig. 8e). Meanwhile, overexpression of MNX1-AS1 significantly decreased miR-6785-5p expression in GC cells (Fig. 8f), whereas miR-6785-5p upregulation failed to cause alteration of MNX1-AS1 expression (Fig. 8g). To further investigate the potential direct binding between lncRNA MNX1-AS1 and miR-6785-5p, RIP assays were performed in GC cells. It was notable that MNX1-AS1 and miR-6785-5p were enriched in immunoprecipitates of Ago2 than those of IgG in both SGC7901 and MGC803 cells (Fig. 8h).

### LncRNA MNX1-AS1 modulates the expression levels of BCL2 through post-transcriptional regulation of miR-6785-5p

**Fig. 9:**
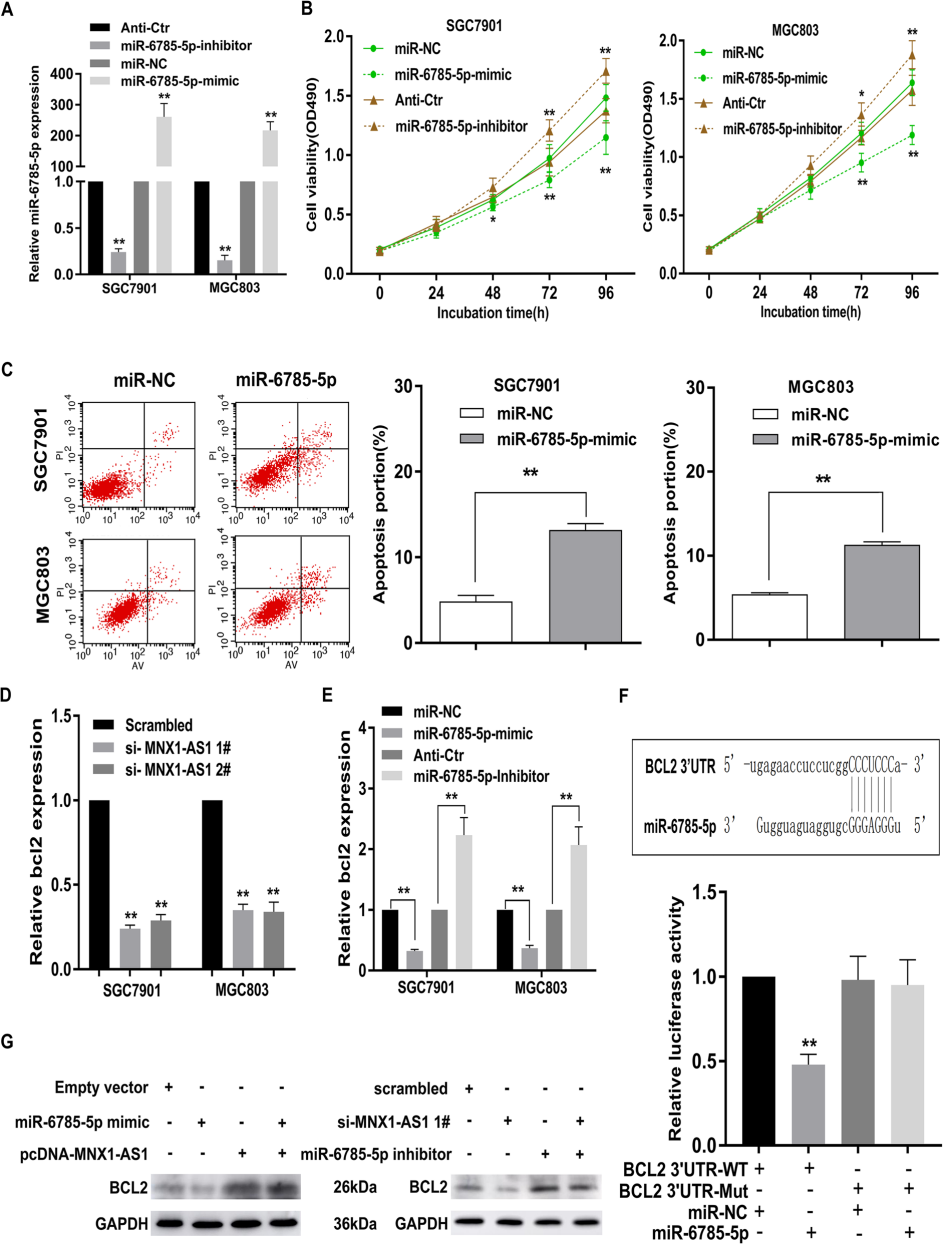
BCL2 is a target of miR-6785-5p and is suppressed by MNX1-AS1 deletion. **a** qRT-PCR assays were used to detect miR-6785-5p in GC cells transfected with miR-6785-5p mimic or miR-6785-5p inhibitor compared with each control group. **b** MTT assays were performed to investigate the effects of altered miR-6785-5p expression on the cell viability in GC. **c** SGC7901 and MGC803 cells transfected with miR-NC or miR-6785-5p were stained and analyzed by cell apoptosis assays. **d** The expression level of BCL2 was remarkably downregulated in GC cells with MNX1-AS1 knockdown. **e** qRT-PCR assays were used to determine the effects of miR-6785-5p dysregulation on BCL2 expression in SGC7901 and MGC803 cells. **f** The prediction result of miR-6785-5p putative targeting site in the WT and Mut 3′ UTR of BCL2. MiR-6785-5p could bind to the predicted site in the 3’UTR of BCL2 mRNA. **g** Western blot assays revealed that miR-6785-5p overexpression can impair the BCL2 upregulation mediated by MNX1-AS1 overexpression, while miR-6785-5p suppression can reduce the BCL2 downregulation induced by MNX1-AS1 knockdown

To further illuminate the biological function of miR-6785-5p in GC, miR-6785-5p mimic or miR-6785-5p inhibitor was used to upregulate or downregulate miR-6785-5p expression in SGC7901 and MGC803 cells (Fig. 9a). MTT assays showed that repression of miR-6785-5p obviously enhanced GC cell viability, while the overexpression of miR-6785-5p led to inverse effects (Fig. 9b). Furthermore, flow cytometric experiments showed that increased miR-6785-5p expression obviously activated apoptosis in GC cells (Fig. 9c).

Interestingly, through RNA-sequencing data analysis, BCL2 was found to be one of the most commonly altered genes (Fig. 6a, b). Consistent with the RNA-sequencing results, the expression level of BCL2 was significantly decreased in GC cells with MNX1-AS1 knockdown at both mRNA and protein levels (Fig. 9d, g)**.** In addition, overexpression or downregulation of miR-6785-5p caused a significant decrease or increase in BCL2 expression at both mRNA and protein levels (Fig. 9e, g). According to these findings, we hypothesized that lncRNA MNX1-AS1, miR-6785-5p and BCL2 might form a common ceRNA network in GC.

**Fig. 10:**
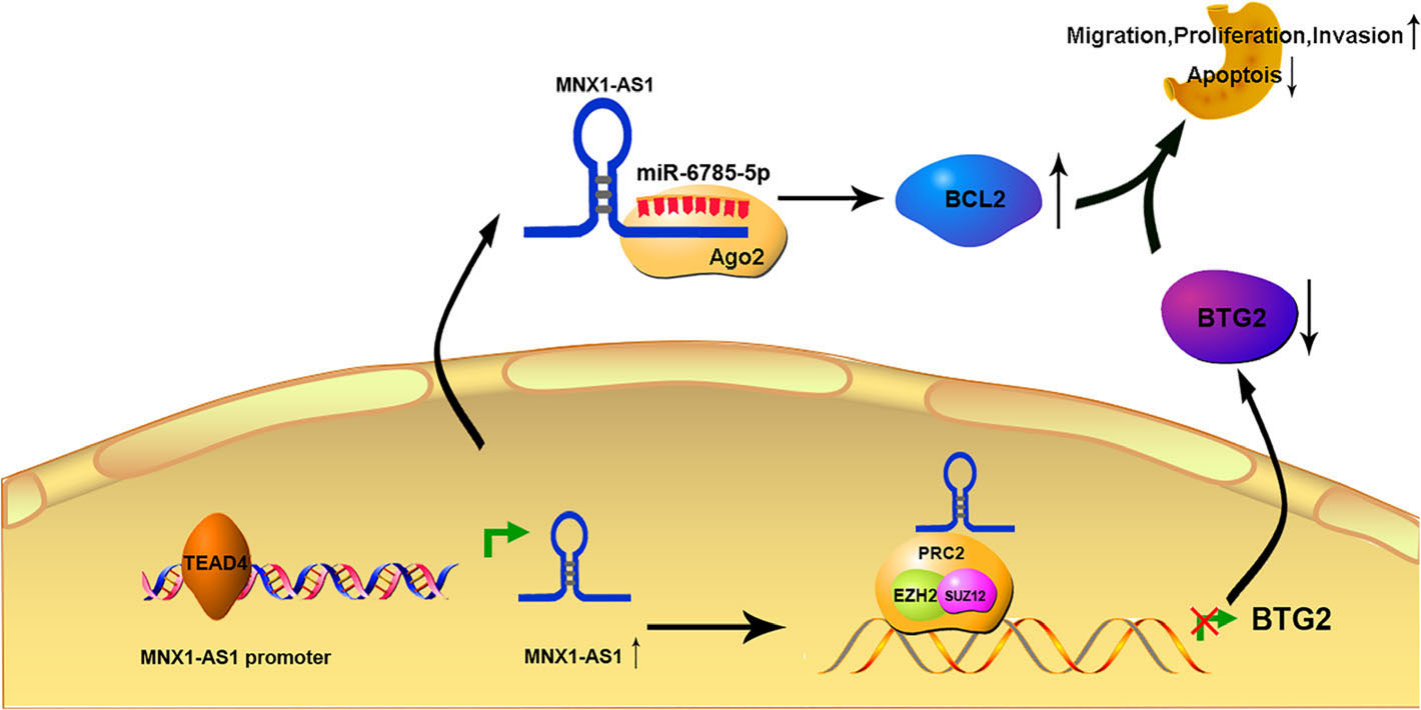
A schematic illustration of the proposed model depicting the molecular mechanism of lncRNA MNX1-AS1 in promoting GC development and progression

To test our hypothesis, we predicted the potential target genes of miR-6785-5p using miRanda and found that BCL2 was likely to bind with miR-6785-5p (Fig. 9f). Indeed, luciferase reporter assays showed that WT-BCL2-driven luciferase expression was significantly inhibited by cotransfection with the miR-6785-5p mimic compared with the control. However, this inhibitory effect was abolished by mutation of the putative miR-6785-5p-binding site in the BCL2 3′ UTR. Taken together, these results indicate that miR-6785-5p can bind to the predicted site in the 3’UTR of BCL2 mRNA and mediated BCL2 expression levels in GC (Fig. 9f). Additionally, we performed rescue assays to evaluate whether lncRNA MNX1-ASl regulates BCL2 by competing for miR-6785-5p. As shown in Fig. 9g, the results showed that overexpression of lncRNA MNX1-AS1 increased BCL2 levels and ectopic expression of miR-6785-5p repressed this increase, whereas suppression of lncRNA MNX1-AS1 decreased BCL2 levels and effects of miR-6785-5p inhibitor impaired this downregulation (Fig. 9g). These findings illuminated the existence of a lncRNA MNX1-AS1-miR-6785-5p-BCL2 regulatory axis. Collectively, BCL2 is a target gene of miR-6785-5p and is indirectly mediated by lncRNA MNX1-AS1 (Fig. 10**).**

## Discussion

A growing body of research has illuminated that dysregulated lncRNAs may mediate cancer progression [41–43]. Recent evidence of the roles of lncRNAs in cancer pathogenesis has added to our knowledge of cancer biology. Although dysregulation of certain lncRNAs in gastric tumourigenesis is a recognized phenomenon, the functional mechanisms of most lncRNAs still remain undetermined in human GC.

Currently, the expression pattern and functional role of lncRNA MNX1-AS1 in diverse tumours have been gradually identified [22, 24, 44, 45] Yang et al. found that overexpression of MNX1-AS1 exerted tumour-promoting roles in lung adenocarcinoma [45]. A current study by Ji et al. showed that enrichment of MNX1-AS1 is strongly linked to TNM stage in hepatocellular carcinoma (HCC) patients [46]. More interestingly, MNX1-AS1 can sponge miR-218-5p to activate the expression of COMMD8, thus facilitating malignant properties of HCC [46]. Previous data provided by Ma et al. showed that impaired MNX1-AS1 expression can regulate CDKN1A expression to suppress invasive capacities of GC cells [24]. To the best of our knowledge, few studies have reported the use of in vitro (let alone in vivo) assays to explore the regulatory correlation between lncRNA MNX1-AS1 and GC, or tried to clarify the upstream regulation of lncRNAs. More studies in epigenetics may shed new light on cancer pathogenesis.

In this study, MNX1-AS1 expression was firstly detected via microarray analysis, and further determined in 174 paired GC tissue samples. Overexpressed MNX1-AS1 promoted GC cell growth and invasion, suggesting its critical value in GC patients. Current evidence has shown that TFs contribute to lncRNA dysregulation in various cancers [25, 47]. TEAD4, a critical member of the TEA domain (TEAD) family, has been identified as an oncogenic regulator in many cancers, including GC [48, 49]. Our data showed that MNX1-AS1 overexpression could be activated by TEAD4.

To clarify the regulatory mechanism of lncRNA MNX1-AS1 in GC, we used RNA-sequencing experiments to investigate potential target genes. It was reported in previous studies that epigenetic alterations such as aberrant histone modifications can participate in cancer development and progression [50–52]. Moreover, lncRNAs have been shown to cooperate with chromatin-modifying enzymes, thus activating epigenetic activation or gene silencing [52]. EZH2, an important member of PRC2, could mediate H3K27me3 and exert oncogenic functions through repressing tumour suppressors [53, 54]. For this study, we found that MNX1-AS1 mainly localized in the nucleus, indicating that MNX1-AS1 may exert its oncogenic effects at transcriptional level. Mechanistic investigations elucidated that lncRNA MNX1-AS1 can recruit EZH2 and H3K27me3 to the promoter of BTG2 in the nucleus, thus partially silencing BTG2 expression and mediating oncogenic properties in GC.

Additionally, we also attach great importance to lncRNA-mediated post-transcriptional processing. For instance, our previous research has reported that linc00346 can function as a ceRNA to sponge miR-34a-5p in GC cells [27]. In the present research, both bioinformatic analyses and luciferase reporter assays demonstrated that lncRNA MNX1-AS1 functions as a ceRNA for miR-6785-5p, thereby contributing to the depression of its endogenous target gene. In addition, miR-6785-5p upregulation impaired GC cell proliferation and induce cell apoptosis, while miR-6785-5p knockdown elicited the opposite effects. Interestingly, both RNA-seq data and subsequent verification assays revealed that BCL2 was obviously downregulated in MNX1-AS1-depleted-GC cells. Prediction results from miRanda also revealed that BCL2 was likely to bind with miR-6785-5p. Thus, we proposed the hypothesis that BCL2 may be a candidate target of miR-6785-5p, To confirm this hypothesis, we used luciferase reporter assays and showed that miR-6785-5p targeted BCL2 mRNA at its 3’UTR.

Collectively, we have uncovered that MNX1-AS1 is a novel lncRNA correlated with GC tumourigenesis and progression. TEAD4, a critical oncogenic transcription factor, may be involved in promoting the transcription of lncRNA MNX1-AS1 via binding to the promoter region of MNX1-AS1. In addition, we identified a molecular mechanism underlying GC development that involves lncRNA MNX1-AS1/EZH2/BTG2 and MNX1-AS1/miR-6785-5p/BCL2 axes (Fig. 10**)**.

## Conclusions

Our findings may facilitate better understanding of GC pathogenesis and shed new light on lncRNA-based diagnosis and treatment of GC and other human malignancies.

## Supplementary information

**Additional file 1: Figure S1**. LncRNA MNX1-AS1 was significantly upregulated in GC cells, and its ectopic expression promoted GC cell migration and invasion. A.The expression level of MNX1-AS1 in GC cells and GES-1 cells. B. The effects of MNX1-AS1 overexpression on GC cell migration and invasion.

**Additional file 2: Table S1**. Univariate and multivariate analysis of clinicopathological factors for overall-survival in gastric cancer patients (*n* = 174).

**Additional file 3: Table S2**. Univariate and multivariate analysis of clinicopathological factors for disease-free survival in gastric cancer patients (n = 174).

**Additional file 4: Table S3**. Primers for qRT-PCR, siRNAs oligonucleotides and the company for antibody.

**Additional file 5:.** Materials and Methods.

## Data Availability

The microarray data used in the study repsitory from TCGA and GEO (GSE62254) datasets. The RNAsequencing raw data and normalized results were submitted to GEO database.

## Abbreviations

BCL2: B-cell lymphoma 2
BTG2: BTG anti proliferation factor 2
CHIP: Chromatin immunoprecipitation assays
ECL: Enhanced chemiluminescence
EZH2: Enhancer of zeste 2
FBS: Fetal bovine serum
FISH: Fluorescence in situ hybridization
GC: Gastric cancer
GEO: Gene Expression Omnibus
H3K27me3: Trimethylation of histone H3 lysine 27
HCC: Hepatocellular carcinoma
IHC: Immunohistochemistry
lncRNAs: Long non-coding RNAs
MAPK: Mitogen-activated protein kinase
mTOR: Mammalian target of rapamycin
PRC2: Polycomb Repressive Complex
qRT-PCR: Quantitative reverse transcription polymerase chain reaction
RBPs: RNA-binding proteins
RISC: RNA-induced silencing complex
TCGA: The Cancer Genome Atlas
TEAD4: TEA domain family member4
TNM: Tumour–node–metastasis
