# Supplementary figures and images for: TEAD4 modulated LncRNA MNX1-AS1 contributes to gastric cancer progression partly through suppressing BTG2 and activating BCL2
Source: Mol Cancer. 2020 Jan 10;19:6. doi: 10.1186/s12943-019-1104-1 (PMC6953272; doi:10.1186/s12943-019-1104-1)

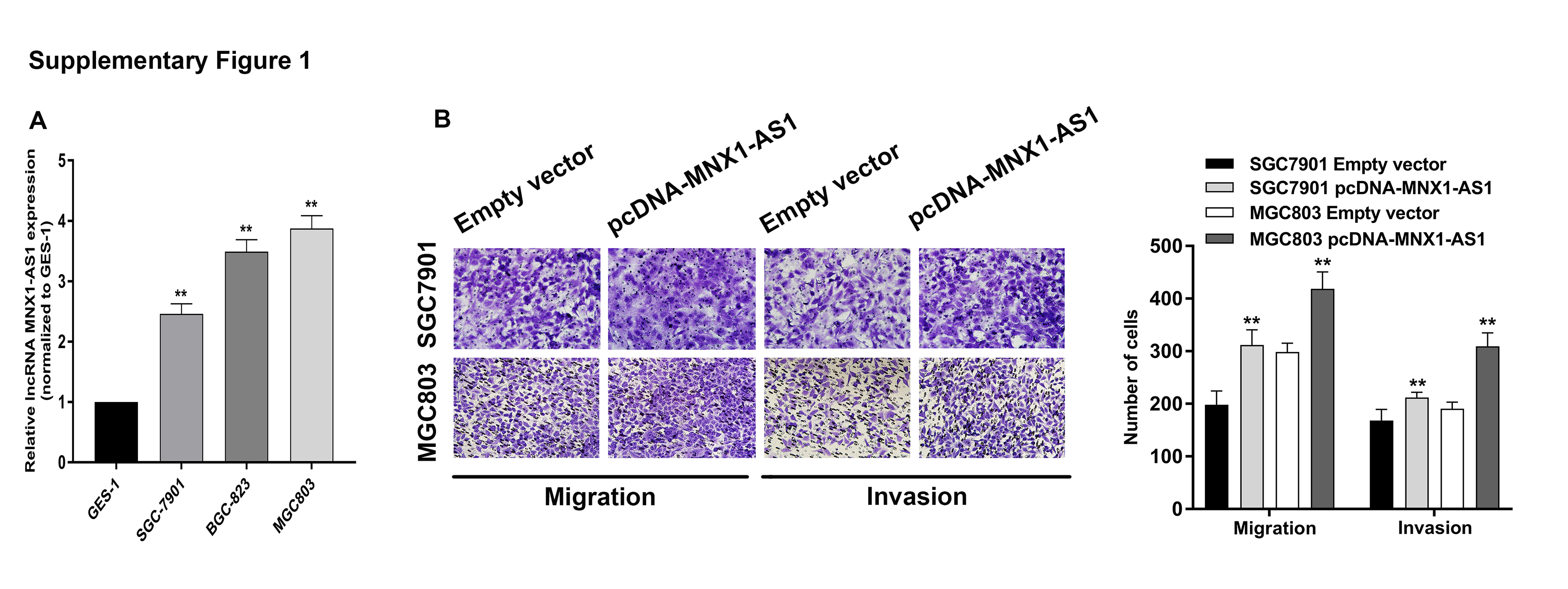

Supplement: Supplementary file 1 — Additional file 1: Figure S1. LncRNA MNX1-AS1 was significantly upregulated in GC cells, and its ectopic expression promoted GC cell migration and invasion. A.The expression level of MNX1-AS1 in GC cells and GES-1 cells. B. The effects of MNX1-AS1 overexpression on GC cell migration and invasion. [file 12943_2019_1104_MOESM1_ESM.tif]
