# Supplementary material for: TEAD4 modulated LncRNA MNX1-AS1 contributes to gastric cancer progression partly through suppressing BTG2 and activating BCL2
Source: Mol Cancer. 2020 Jan 10;19:6. doi: 10.1186/s12943-019-1104-1 (PMC6953272; doi:10.1186/s12943-019-1104-1)
Supplement: Supplementary file 2 — Additional file 2: Table S1. Univariate and multivariate analysis of clinicopathological factors for overall-survival in gastric cancer patients (n = 174). [file 12943_2019_1104_MOESM2_ESM.docx]

**Table S1.** Univariate and multivariate analysis of clinicopathological factors for overall-survival in gastric cancer patients (n=174).

| **Variables** | **Univariate analysis** | | | **Multivariate analysis** | | |
| --- | --- | --- | --- | --- | --- | --- |
|  | HR | 95% CI | p value | HR | 95% CI | p value |
| **Age**  (≤50/ >50) | 1.751 | 0.701-4.376 | 0231 |  |  |  |
| **Gender**  (Male/Female) | 0.996 | 0.555-1.786 | 0.988 |  |  |  |
| **Tumor size**  (<5/≥5) | 2.015 | 1.201-3.380 | 0.008* | 1.418 | 0.778-2.582 | 0.254 |
| **Location**  (middle + proximal vs distal) | 0.855 | 0.515-1.419 | 0.544 |  |  |  |
| **Histologic grade**  (Well and moderate VS Poor) | 1.695 | 0.984-2.922 | 0.057 |  |  |  |
| **Depth of tumor**  (T3, T4 / T1, T2) | 2.578 | 1.224-5.429 | 0.013* | 0.597 | 0.238-1.497 | 0.271 |
| **Lymphatic metastasis**  (no vs yes) | 4.332 | 2.053-9.141 | <0.001* | 1.880 | 0.712-4.963 | 0.203 |
| **Distant metastasis**  (no vs yes) | 5.526 | 2.769-11.030 | <0.001* | 3.173 | 1.415-7.114 | 0.005* |
| **TNM stage**  (III+IV vs I+II) | 5.182 | 2.936-9.144 | <0.001* | 2.781 | 1.244-6.214 | 0.013* |
| **MNX1-AS1 expression**  (high vs low) | 3.362 | 1.930-5.854 | <0.001* | 2.513 | 1.370-4.610 | 0.003* |

HR, hazard ratio; 95 % CI, 95 % confidence interval, * Overall *P* < 0.05.
