# Supplementary material for: TEAD4 modulated LncRNA MNX1-AS1 contributes to gastric cancer progression partly through suppressing BTG2 and activating BCL2
Source: Mol Cancer. 2020 Jan 10;19:6. doi: 10.1186/s12943-019-1104-1 (PMC6953272; doi:10.1186/s12943-019-1104-1)
Supplement: Supplementary file 3 — Additional file 3: Table S2. Univariate and multivariate analysis of clinicopathological factors for disease-free survival in gastric cancer patients (n = 174). [file 12943_2019_1104_MOESM3_ESM.docx]

**Table S2.** Univariate and multivariate analysis of clinicopathological factors for disease-free survival in gastric cancer patients (n=174).

| Variables | Univariate analysis | | | Multivariate analysis | | |
| --- | --- | --- | --- | --- | --- | --- |
|  | HR | 95% CI | p value | HR | 95% CI | p value |
| **Age**  (≤50/ >50) | 1.085 | 0.539-2.183 | 0.819 |  |  |  |
| **Gender**  (Male/Female) | 1.103 | 0.640-1.901 | 0.723 |  |  |  |
| **Tumor size**  (<5/≥5) | 1.788 | 1.118-2.858 | 0.015* | 1.239 | 0.721-2.127 | 0.438 |
| **Location**  (middle + proximal vs distal) | 0.906 | 0.571-1.439 | 0.676 |  |  |  |
| **Histologic grade**  (Well and moderate VS Poor) | 1.756 | 1.063-2.900 | 0.028* | 1.737 | 1.039-2.907 | 0.035* |
| **Depth of tumor**  (T3, T4 / T1, T2) | 2.824 | 1.404-5.683 | 0.004* | 0.912 | 0.396-2.101 | 0.829 |
| **Lymphatic metastasis**  (no vs yes) | 4.471 | 2.218-9.011 | <0.001* | 2.321 | 0.975-5.525 | 0.057 |
| **Distant metastasis**  (no vs yes) | 3.973 | 2.015-7.835 | <0.001* | 2.333 | 1.085-5.019 | 0.030* |
| **TNM stage**  (III+IV vs I+II) | 4.442 | 2.672-7.385 | <0.001* | 2.147 | 1.079-4.269 | 0.029* |
| **MNX1-AS1 expression**  (high vs low) | 3.062 | 1.861-5.040 | <0.001* | 2.266 | 1.317-3.899 | 0.003* |
