# Supplementary material for: TEAD4 modulated LncRNA MNX1-AS1 contributes to gastric cancer progression partly through suppressing BTG2 and activating BCL2
Source: Mol Cancer. 2020 Jan 10;19:6. doi: 10.1186/s12943-019-1104-1 (PMC6953272; doi:10.1186/s12943-019-1104-1)
Supplement: Supplementary file 4 — Additional file 4: Table S3. Primers for qRT-PCR, siRNAs oligonucleotides and the company for antibody. [file 12943_2019_1104_MOESM4_ESM.docx]

**Table S3. Primers for qRT-PCR, siRNAs oligonucleotides and the company for antibody.**

| **Primers used for qRT-PCR** |  |
| --- | --- |
| TEAD4-F | GGACACTACTCTTACCGCATCC |
| TEAD4-R | TCAAAGACATAGGCAATGCACA |
| GAPDH-F | GCTCTCTGCTCCTCCTGTTC |
| GAPDH-R | ACGACCAAATCCGTTGACTC |
| BTG2-F | CATCATCAGCAGGGTGGC |
| BTG2-R | CCCAATGCGGTAGGACAC |
| MNX1-AS1-F | AAGGTAGCCACCAAACAC |
| MNX1-AS1-R | AGACTCACGTAGCACTGT |
| EZH2-F | TGCACATCCTGACTTCTGTG |
| EZH2-R | AAGGGCATTCACCAACTCC |
| BCL2-F | GCACAAATACTCCGCAAG |
| BCL2-R | GAGAGAATGTTGGCGTCT |
| **ChIP primers** |  |
| TEAD4-MNX1-AS1-F | TTTCCCCGGTGATAAGCCCT |
| TEAD4-MNX1-AS1-R | GCCTTGCCTTCTCTTCCTCTC |
| EZH2-BTG2-F | GCTGACCTTTCTGGACGGAG |
| EZH2-BTG2-R | CCACCTCAATGTCAGCCGAA |
| **siRNAs oligonucleotides** |  |
| si-MNX1-AS1 1# | AGGUAGCCACCAAACACAUGCAUAA |
| si-MNX1-AS1 2# | GAGUCUUGCAAAGAGGAGAUCUUUA |
| si-MNX1-AS1 3# | CAUACAACUCGACAGAGUCACAGAA |
| siTEAD4 1# | GTATGCTCGCTATGAGAAT |
| siTEAD4 2# | GGACATCCGCCAAATCTAT |
| siTEAD4 3# | GAAGCCTTTCTCTCAGCAA |
| **Antibody** | **Company** |
| GAPDH | Cell Signaling Technology |
| TEAD4 | Abcam |
| BTG2 | Abcam |
| IgG | Millipore |
| EZH2 | Millipore |
| SUZ12 | Millipore |
| Ago2 | Millipore |
| BCL2 | Genetex |
| H3K27me3 | Abcam |
| **FISH probe sequences** |  |
| LncRNA MNX1-AS1 | CGATGGACGCCACCTTCTGTGACTCTGT |
