# Supplementary material for: TEAD4 modulated LncRNA MNX1-AS1 contributes to gastric cancer progression partly through suppressing BTG2 and activating BCL2
Source: Mol Cancer. 2020 Jan 10;19:6. doi: 10.1186/s12943-019-1104-1 (PMC6953272; doi:10.1186/s12943-019-1104-1)
Supplement: Supplementary file 5 — Additional file 5:. Materials and Methods. [file 12943_2019_1104_MOESM5_ESM.doc]

**Supplementary Materials and Methods**

**Cell migration and invasion assays**

For migration assays, 5 x 10^4^ transfected GC cells were placed in the upper chamber of an insert. For the invasion experiments, the top side of the upper chamber membrane of the insert was coated with Matrigel and DMEM or RPMI 1640 medium containing 10% FBS was used to fill the upper chamber. The lower culture dish contained medium supplemented with 20% FBS. After 24-48 hours, we removed the cells remaining on the upper membrane with cotton wool. At the same time, the cells on the other side of the membrane were fixed with methanol and stained with 0.1% crystal violet solution. Finally, a microscope was used to observe and count cells. The experiment was repeated three times independently.

**Cell proliferation assay**

Cell proliferation ability was monitored using a Proliferation Reagent Kit I (MTT) (Roche, Basel, Switzerland). The cells were seeded in 96-well plates, and cell viability was recorded every 24 hours according to the manufacturer's instructions. For colony formation assays, the cells were grown for 10 days in a six-well plate in a suitable medium containing 10% FBS. Colonies were fixed with methanol and stained with 0.1% crystal violet.

**Flow cytometry assay**

We analysed cell cycle and apoptosis using flow cytometry assays. GC cells were harvested at 48 hours after transfection. For apoptosis assays, harvested cells were double-stained with propidium iodide (PI) according to the protocol of a FITC-Annexin V cell apoptosis assay kit (BD Biosciences). The cells were then analysed using a flow cytometer (FACScan; BD Biosciences) equipped with CellQuest software (BD Biosciences). Living cells, dead cells, early apoptotic cells and apoptotic cells were distinguished, with the focus laid on the relative proportion of early and late apoptotic cells. For cell cycle assays, the harvested cells were stained with PI using a CycleTEST PLUS DNA kit (BD Biosciences). The percentage of cells in the phase of G0/G1, S or G2/M was calculated and compared between the transfected and control groups according to FACScan analysis.

**Tumour formation assay**

We maintained 4-week-old male athymic BALB/c nude mice under specific pathogen-free (SPF) conditions. MGC-803 cells stably transfected with the sh-MNX1-AS1 or scrambled construct were harvested and resuspended at a density of 2*10^7^ cells/ml. Then, 100 μl of the shRNA- or scramble-transfected cells were subcutaneously injected into either side of each mouse, respectively. Tumour growth was checked every 3 days. Tumour volume was calculated using the following equation: V=0.5*D*d^2^ (V, volume; D, longitudinal diameter; d, latitude diameter). Seventeen days after injection, the mice were euthanized and tumours were stripped for further analysis (weight measurement, RNA extraction and IHC).

**RNA immunoprecipitation(RIP)**

An EZMagna RNA Immunoprecipitation (RIP) Kit (Millipore) was used according to the manufacturer's protocol. First, SGC7901 and MGC803 cells were lysed in complete RIP lysis buffer. The extract was then mixed with magnetic beads conjugated with a specific antibody or control IgG (Millipore) and incubated at 4 °C for 6-8 hours. Next, to remove the protein, the beads were washed with wash buffer and incubated with proteinase K for 30 minutes at 55 °C. Finally, the purified RNA was subjected to qRT-PCR analysis. All antibody information is listed in **Supplementary Table 3.**

**Chromatin immunoprecipitation assays(ChIP)**

Chromatin immunoprecipitation (ChIP) experiments were performed using a MagnaChIP kit (Millipore) according to the manufacturer's instructions. Incubation of SGC7901 and MGC803 cells with 4% paraformaldehyde for 10 minutes resulted in DNA-protein cross-linking. The cells were lysed with cell lysate and sonicated to produce 200-300 bp chromatin fragments. Next, the lysate was immunoprecipitated with magnetic protein A beads conjugated with TEAD4, EZH2 or H3K27me3, with normal mouse IgG used as a control. Finally, the precipitated DNA was analysed via qRT-PCR assay. The primers were listed in Supplementary Table 3.

**Luciferase assays**

Luciferase assays were performed using a luciferase assay kit (Promega, Madison, WI, USA) based on the manufacturer's protocol. The TEAD4 binding sequence on the promoter region of MNX1-AS1 was determined by JASPAR (http://jaspar.genereg.net/), and different fragment sequences were synthesized, and then inserted into a pGL3-basic vector. A complementary DNA fragment containing the wild type or mutant MNX1-AS1 fragment and the 3’untranslated region (UTR) of BCL2 was subcloned downstream of the luciferase gene in a pMIR-GLO-luciferase reporter vector. Luciferase assays were evaluated using the dual luciferase kit (Promega). Relative firefly luciferase activity was normalized to Renilla luciferase activity. Each experiment was repeated three times.

**Fluorescence in situ hybridization (FISH) and subcellular separation**

The nuclei and cytoplasm of SGC-7901 and MGC-803 cells were isolated using a PARIS kit (Life Technologies) according to the manufacturer's protocol. GC cells were fixed in 4% formaldehyde for 15 minutes and then washed with PBS. The fixed cells were treated with pepsin (1% in 10 mmol/L HCl) and then dehydrated in 70%, 90% and 100% ethanol. The air-dried cells were further incubated with a 40 nmol/L FISH probe in hybridization buffer (100 mg/mL dextran sulphate, 10% formamide in 2XSSC) for 2 minutes at 80 ℃. Hybridization was carried out at 55℃ for 2 hours, and then, the cells on the slide were washed and dehydrated. Air-dried slides were tested with Prolong Gold Antifade Reagent using DAPI. The probe sequences are listed in the **Supplementary Table 3.**
